# Feline tetherin (BST-2) restricts feline immunodeficiency virus release but not spreading infection

**DOI:** 10.1186/1742-4690-8-S2-O10

**Published:** 2011-10-03

**Authors:** Isabelle Dietrich, Elizabeth L McMonagle, Sarah J Petit, Swetha Víjayakríshnan, Nicola Logan, Chi N Chan, Greg J Towers, Margaret J Hosie, Brian J Willett

**Affiliations:** 1 MRC-University of Glasgow Centre for Virus Research, University of Glasgow, Glasgow, UK; 2 MRC Centre for Medical Molecular Virology, University College London, London, UK

## Background

Tetherin (BST-2) is an interferon-inducible transmembrane protein that inhibits the release of enveloped viruses from infected cells. Here, we characterise the feline homologue of tetherin and assess its effects on the replication of feline immunodeficiency virus (FIV).

## Results

A feline homologue of tetherin was amplified from IL2-dependent T cell cDNA. Real-time PCR analyses revealed that feline tetherin transcripts were expressed in many feline cell lines and were induced by interferons (α, ω or γ). Like human tetherin, feline tetherin displayed potent inhibition of FIV and HIV-1 particle release, however, this activity resisted antagonism by either HIV-1 VPU, or the FIV Env and "OrfA" proteins. Further, as over-expression of a complete FIV genome in trans could not overcome feline tetherin, these data suggest that FIV lacks a functional tetherin antagonist. When expressed stably in feline cell lines, tetherin did not abrogate the replication of FIV, indeed, syncytia formation was significantly enhanced in tetherin-expressing cells infected with CD134-independent strains of FIV (FIV Fca-F14 and FIV Pco-CoLV). Confocal microscopy revealed co-localisation of tetherin and FIV envelope glycoprotein (Env) in regions at the periphery of syncytia. Moreover, while treatment of feline cells with ínterferon-ω suppressed viral protein production and release (consistent with the pleiotropic actions of type-l interferon on viral replication), syncytia formation was enhanced in cells treated with interferon-ω either pre- or post- infection with a CD134-independent strain of FIV.

## Conclusions

While feline tetherin prevents the release of nascent viral particles, cell to cell spread remains efficient in the presence of abundant viral receptors and interferon-induced up-regulation of tetherin expression may enhance syncytia formation. Accordingly, tetherin expression *in vivo* may promote the selective expansion of viral variants capable of more efficient cell to cell spread; contributing to the emergence of CD134-independent strains of FIV.

